# Bacterial Communities in the Gut and Reproductive Organs of *Bactrocera minax* (*Diptera: Tephritidae*) Based on 454 Pyrosequencing

**DOI:** 10.1371/journal.pone.0106988

**Published:** 2014-09-12

**Authors:** Ailin Wang, Zhichao Yao, Weiwei Zheng, Hongyu Zhang

**Affiliations:** State Key Laboratory of Agricultural Microbiology, Institute of Urban and Horticultural Pests, and Hubei Insect Resources Utilization and Sustainable Pest Management Key Laboratory, College of Plant Science and Technology, Huazhong Agricultural University, Wuhan, China; University of Aveiro, Portugal

## Abstract

The citrus fruit fly *Bactrocera minax* is associated with diverse bacterial communities. We used a 454 pyrosequencing technology to study in depth the microbial communities associated with gut and reproductive organs of *Bactrocera minax*. Our dataset consisted of 100,749 reads with an average length of 400 bp. The saturated rarefaction curves and species richness indices indicate that the sampling was comprehensive. We found highly diverse bacterial communities, with individual sample containing approximately 361 microbial operational taxonomic units (OTUs). A total of 17 bacterial phyla were obtained from the flies. A phylogenetic analysis of 16S rDNA revealed that *Proteobacteria* was dominant in all samples (75%–95%). *Actinobacteria* and *Firmicutes* were also commonly found in the total clones. *Klebsiella*, *Citrobacter*, *Enterobacter*, and *Serratia* were the major genera. However, bacterial diversity (Chao1, Shannon and Simpson indices) and community structure (PCA analysis) varied across samples. Female ovary has the most diverse bacteria, followed by male testis, and the bacteria diversity of reproductive organs is richer than that of the gut. The observed variation can be caused by sex and tissue, possibly to meet the host's physiological demands.

## Introduction

Insects harbor diverse bacterial communities in their digestive systems [1]. Most intestinal bacteria are benign or beneficial to insects [2]. They play an array of important roles in interactions with their insect hosts, many of which are nutritional functions. For example, the bacteria *Buchnera* can provide aphids with essential amino acids [3]. Recent studies have shown that high levels of *Pseudomonas aeruginosa* can significantly reduce the longevity of the medfly [4]. In addition to providing nutrients, bacteria can increase the fitness of insects through other mechanisms [5], such as protecting the insects from pathogenic viruses and parasites [6]–[8] and improving the insect's tolerance to heat stress [9]. Some interactions between hosts and microbes have been well studied. However, the exact nature of how bacterial communities are structured; and the functions of each species are still unclear.

Early studies on insect bacterial diversity were mostly based primarily on cultivation methods [10], [11]. However, a large number of bacteria are unculturable [12]. In recent years, advances in molecular techniques have provided a new strategy for characterizing all microbes in insects. For example, by using denaturing gradient gel electrophoresis (DGGE), the microbial composition of the intestinal tract of locusts and beetles has been successfully explored [13], [14]. Based on 16S rRNA sequence data, the composition of bacterial communities in a laboratory-scale nitrification reactor and a wastewater treatment plant was detected by 454-pyrosequencing [15].

Fruit flies (*Diptera: Tephritidae*) are important agricultural pests due to their capacity to oviposit in fruits [5]. Previous studies have shown that gut bacteria have important functions in fruit flies, such as *Ceratitis capitata* [5], [16] and *Bactrocera dorsalis* Hendel [17], [18]. The citrus fruit fly *Bactrocera (Tetradacus) minax (Enderlein)* is a destructive pest of citrus fruits in China [19]–[21]. However, the composition of bacterial communities harboured by *B.minax* is still unclear.

In this study, we used the barcoded 454-pyrosequencing to investigate bacterial genomics in the gut and reproductive organs of *B. minax*, in order to characterize the composition and diversity of the microbial communities in the *B. minax* and compare microbial communities associated with different sexes and tissues. Our results will help to understand the importance of symbiotic bacteria for *B. minax.*

## Results

### Pyrosequencing data

The bacteria in *B. minax* were quantified by 454 pyrosequencing of 16S rDNA gene amplicons. We obtained 100749 high quality pyrosequencing reads with an average read length of 400 bp of the 16S rDNA spanning the variable regions V3 and V4 from our samples. These reads were distributed among the samples as follows: male gut (21.90%), male testis (30.52%), female gut (23.40%) and female ovary (24.19%), and yielded 322 OTUs, 319 OTUs, 415 OTUs and 389 OTUs, respectively (Table 1) at the 97% identity threshold.

**Table 1:** Richness and diversity estimation of the 16S rRNA gene libraries from the pyrosequencing analysis.

Sample^a^	Number of reads	Number of OTUs^b^	ACE	Chao1	Shannon	Simpson	Coverage
FI	7857	319	670.080	532.206	3.217	11.90	98.26%
FR	8124	415	728.423	635.037	3.616	13.26	98.09%
MI	7353	322	766.939	566.026	3.162	12.55	97.97%
MR	8957	389	762.483	629.730	3.565	12.73	98,32%

a sample was named according to the gender and tissue.

b OTUs were defined based on 3% sequence divergence.

Abbreviations: FI, female-intestine; FR, female-ovary; MI, male-intestine; MR, male-testis. There are three replicates for each type of sample.

**Figure 1:**
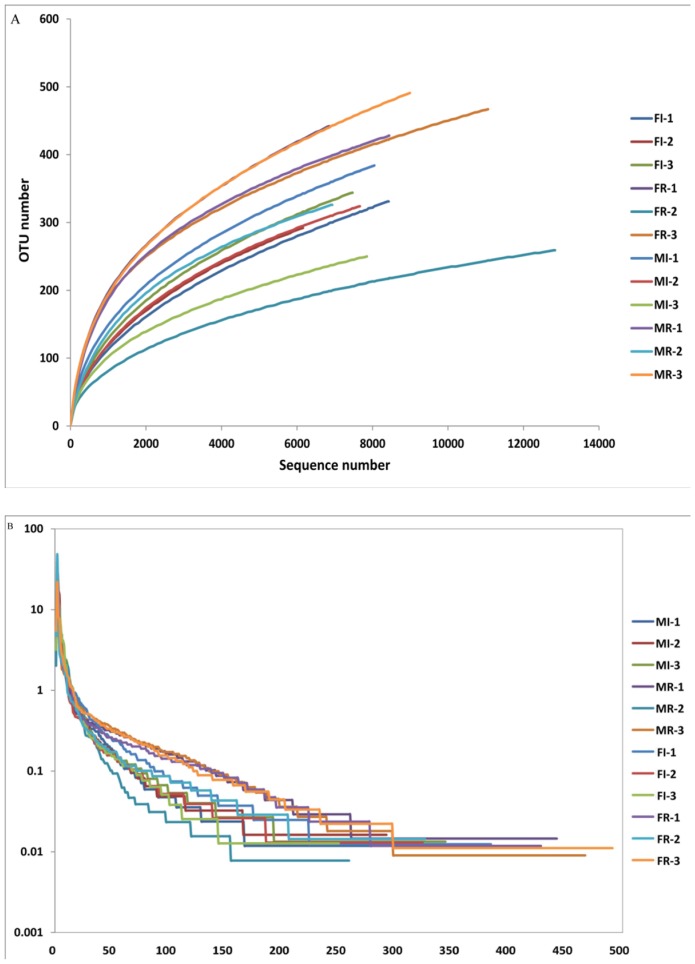
Diversity of bacterial communities in each sample. (A) Rarefaction curve and (B) rank-abundance curve based on bacterial OTUs at a dissimilarity level of 3%. Abbreviations: FI, female-intestine; FR, female-ovary; MI, male-intestine; MR, male-testis. The numbers 1, 2 and 3 represented the three replicates for each type of sample.

The number of bacteria species detected in a sample was strongly affected by the numbers of sequences analyzed [18]. We calculated the rarefaction curves at a 97% similarity level to verify whether the amount of sequencing reflected the diversity of the original microbial communities. The rarefaction analysis revealed that estimates of OTUs sharply increased before approaching a plateau. Our results showed that all bacterial libraries from our samples represented the bacterial communities well, as the rarefaction curves tended towards saturation (Figure 1A). The percentage coverage of FI, FR, MI and MR was 98.3%, 98.1%, 98.0% and 98.3%, respectively (Table 1). Rank-abundance curves indicate that a majority of reads belonged to rare organisms, whereas all samples contained relatively low proportions of highly abundant bacteria (Figure 1B).

We compared the compositions of OTUs and the relative abundance of sequences obtained from each OTU for every sample to calculate the similarity among the samples. The cluster analysis indicates that the bacterial communities in replicate samples were highly similar (Figure S1). Samples from different sexes were grouped into one large cluster, with no clear distinction between sexes. However, samples from different tissues were grouped into two large clusters, and those from the gut were more similar to each other than those from the reproductive organs.

### Taxonomic composition of bacteria identified by pyrosequencing

**Figure 2:**
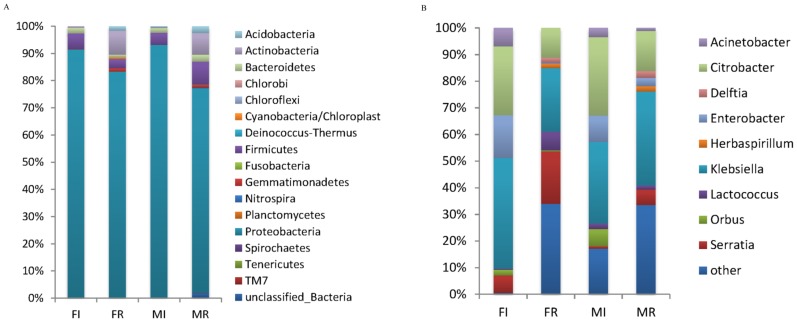
Taxonomic classification of bacterial reads retrieved from different samples with a confidence threshold of 50%. (A) Relative abundance of bacterial at phylum level. (B) Relative abundance of top 15 bacterial genera. Abbreviations: FI, female-intestine; FR, female-ovary; MI, male-intestine; MR, male-testis.

Altogether, 17 bacteria phyla were detected in our samples. The relative abundances of different bacterial groups in each bacterial library were shown in Figure 2A. *Proteobacteria* was dominant in all of the libraries, composing 91.45%, 92.86%, 75.3% and 82.2% of the bacterial communities in the male gut, female gut, testis, and ovary, respectively, followed by *Firmicutes*, composing 4.42%, 5.80% and 8.48% in the male gut, female gut and male testis. Besides, *Actinobacteria* was also a major phylum in male testis and (7.97%) female ovary (8.64%). Reads belonging to *Chlorobi, Chloroflexi, Fusobacteria, Deinococcus-Thermus, Nitrospira, Planctomycetes, Spirochaetes, Tenericutes* and TM7 were found to be minor groups, as they appeared in various libraries with only a few reads (<1%). Another 5 groups including *Acidobacteria, Bacteroidetes, Cyanobacteria/Chloroplast, Gemmatimonadetes* and unclassified bacteria were recovered from our samples.

**Figure 3:**
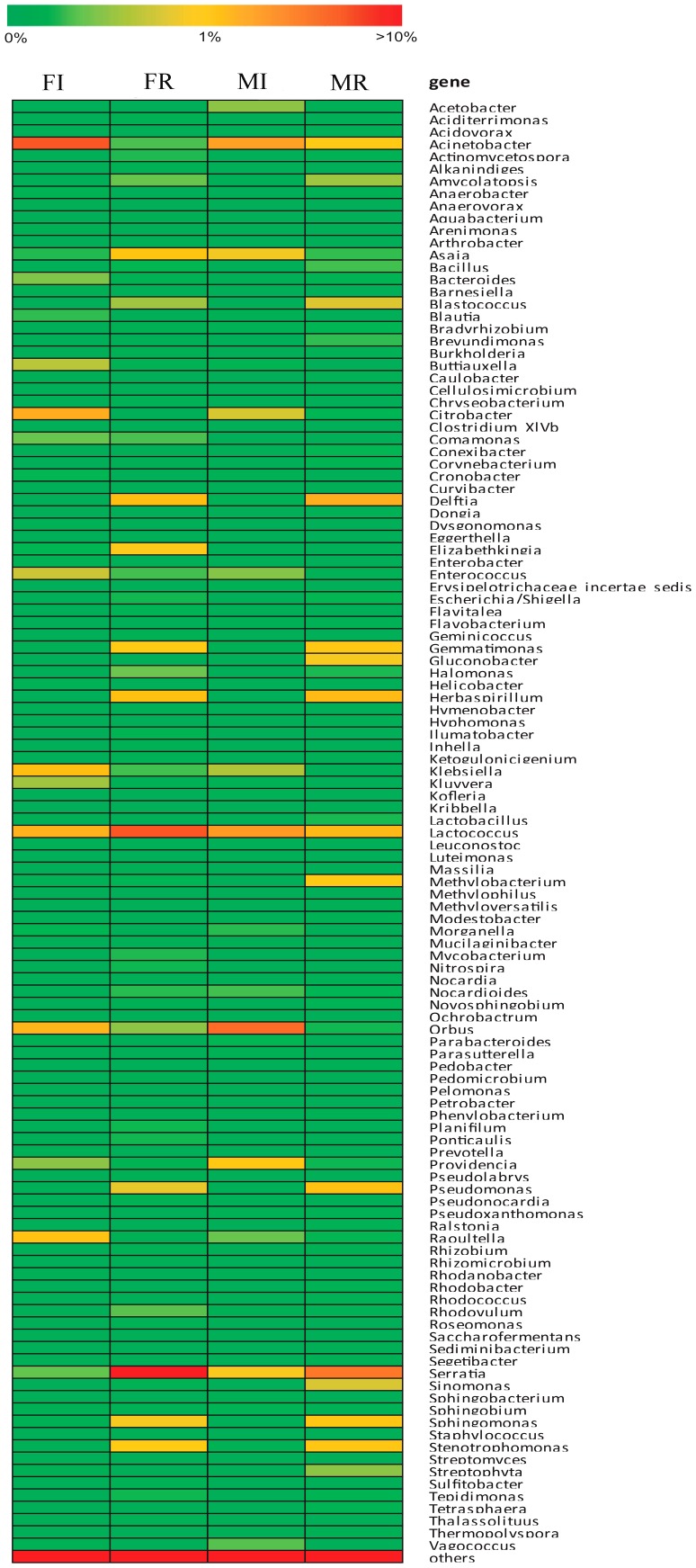
Heatmap showing the relative abundance and distribution of 16S rRNA tag sequences assigned into selected clusters. The color code indicates relative abundance, ranging from green (low abundance) to yellow to red (high abundance). Abbreviations: FI, female-intestine; FR, female-ovary; MI, male-intestine; MR, male-testis.

The relative abundance and distribution of the bacteria from each sample at genus level are shown in Figure 2B and Figure 3. *Klebsiella* constituted a dominant population in all samples, with 41.41% in female gut, 30.52% in male gut, 23.78% in female ovary and 35.28% in male testis. Other major bacteria corresponded to *Citrobacter* with 25.80% in female gut, 29.41% in male gut and 15.09% in male testis. But *Serratia* (19.53%) was strongly represented in female ovary.

The OUT sequences have been submitted to NCBI GenBank (KJ780722–KJ780731 and KM058914–KM061009). The blast results of the 10 most abundant OTUs were shown in table 2. The three species dominating most samples corresponded to *Klebsiella*, *Citrobacter* species: *Klebsiella oxytoca, Raoultella terrigena* and *Citrobacter werkmanii*. The relative abundance of these taxa varied with sexes and tissues. *K. oxytoca* dominated the male testis (34.01%) and showed a relatively high abundance in female gut (16.42%) and male gut (17.77%); *R. terrigena* accounted for 22.5% in male gut and 16.88% in female gut; *C. werkmanii* was strongly represented in both male gut (17.50%) and female gut (16.63%) (Table 3).

**Table 2:** Blast of the 10 most abundant OTUs in the NCBI database.^a^

OUT	Accession	BLAST top hit	Score	E value	%ID
OTU1	KJ780722	*Klebsiella oxytoca*s strain Au04	560	3e-156	99
OTU2	KJ780723	Raoultella terrigena strain 2w5	767	0	99
OTU3	KJ780724	Citrobacter werkmanii strain BF-6	599	7e-168	96
OTU4	KJ780725	Citrobacter freundii strain R2A5	774	0	99
OTU5	KJ780726	Enterobacter cloacae strain SNCE 10	771	0	99
OTU6	KJ780727	Serratia marcescens strain S4	483	5e-133	99
OTU7	KJ780728	Citrobacter murliniae strain L_43	752	0	99
OTU8	KJ780729	Enterobacter aerogenes strain K_G_AE-4	484	1e-133	99
OTU9	KJ780730	Lactococcus lactis subsp.lactis strain SD1S3L10	484	1e-133	100
OTU10	KJ780731	Serratia grimesii strain FA2	621	1e-174	100

a Blast was performed based on 16S rRNA gene.

**Table 3:** Abundance of the major 10 speices in four samples, expressed as % of total in each sample.

Speices	FI	FR	MI	MR
*Klebsiella oxytoca*	16.42	17.77	3.10	34.01
*Raoultella terrigena*	16.88	3.66	22.55	0
*Citrobacter werkmanii*	16.63	0	17.50	2.09
*Citrobacter freundii*	3.63	5.45	4.33	7.19
*Enterobacter cloacae*	4.84	0	9.72	1.10
*Serratia marcescens* subsp. *Sakuensis*	0	14.03	0	0
*Citrobacter murliniae*	0	2.68	3.53	4.64
*Enterobacter soli*	10.20	0	0	1.97
*Lactococcus lactis*	0	7.07	2.12	1.54
*Serratia grimesii*	0	2.26	0	4.55

Abbreviations: FI, female-intestine; FR, female-ovary; MI, male-intestine; MR, male-testis.

### The influences of sex and tissue on bacterial communities in *B.minax*

**Figure 4:**
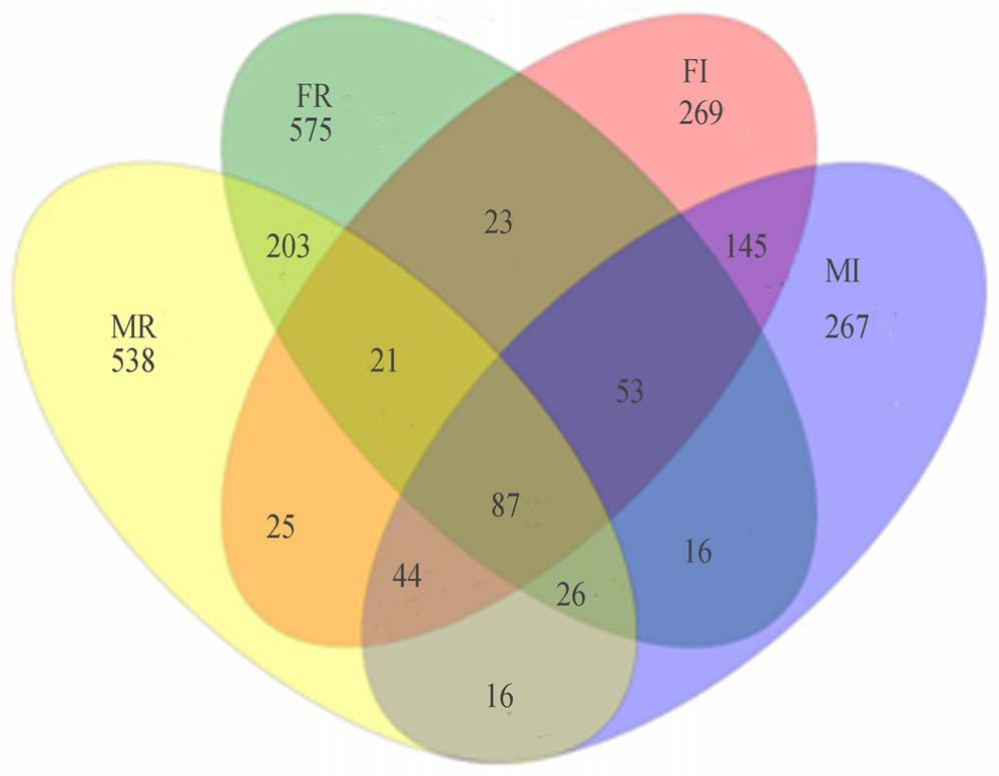
Venn diagram at distance 0.03. The numbers represent the the number of unique species owned by each sample and common species shared by two or more samples. Abbreviations: FI, female-intestine; FR, female-ovary; MI, male-intestine; MR, male-testis.

**Figure 5:**
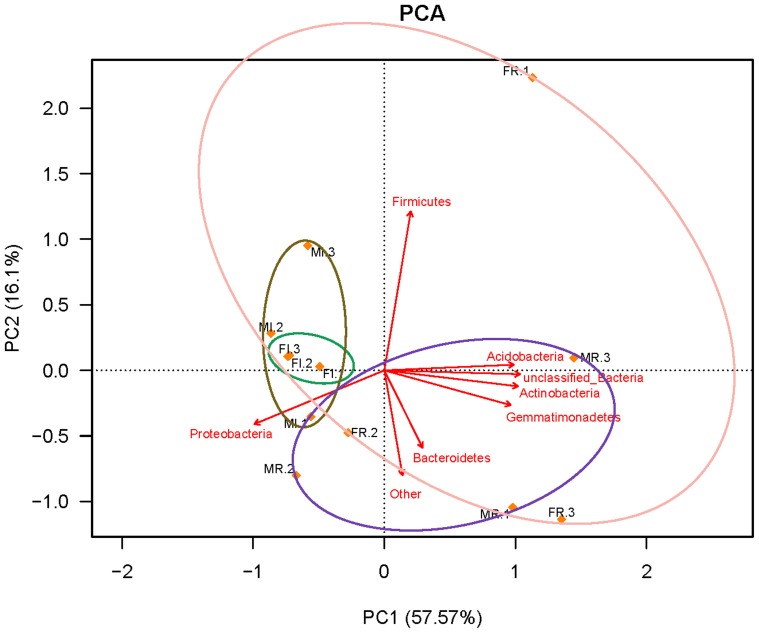
Principal Component Analysis of individual sample and relative abundance of bacterial groups in each sample. Abbreviations: FI, female-intestine; FR, female-ovary; MI, male-intestine; MR, male-testis.

We compared the four samples to find out the influences of sex and tissue on bacterial communities in *B. minax*. Our results showed that the numbers of species in the female (667 in the gut and 1004 in the ovary) were similar to that in the male (654 in the gut and 960 in the testis) (Figure 4), and the abundance of major species was almost the same between them (Table 3 and Figure 3). When compared the bacterial communities in different tissues from the same sex we found that the reproductive organs had higher richness and diversity index (Table 1) and more bacteria species than the gut (Figure 4). At phylum level, *Proteobacteria* was dominant among all samples but more abundant in the gut than in the reproductive organs. Besides, most phyla had significantly different abundance among the four samples (Table 4). Furthermore, there were differences at genus level. Some genera such as *Delftia* and *Herbaspirillum* were only represented in reproductive organs. Most genera had different abundance among different samples. Some genera were rich in one sample but very low in another (Figure 2B and Figure 3). The PCA analysis showed a distinct clustering among the individual samples and the reproductive organ samples became more dispersed than the gut samples (Figure 5). The PCA yielded two main axes that accounted for 73.67% of the total variation in bacterial community structure (Figure 5). This result indicates the influence of the different variables on the various bacterial phylotypes. The reproductive organ samples were correlated with the presence of most phyla. However, correlations were detected between gut samples and the major phylum, *Proteobacteria* (Figure 5).

**Table 4:** Comparisons of the abundance of gut bacteria from the four samples at phylum level.

Phylum	MI	MR	FI	FR
*Acidobacteria*	0.34±0.04^c^	2.49±0.10^a^	0^d^	1.58±0.07^b^
*Actinobacteria*	2.42±0.03^b^	7.97±0.17^b^	0.47±0.05^c^	8.64±0.16^a^
*Bacteroidetes*	1.78±0.09^c^	2.50±0.10^a^	2.16±0.10^b^	1.02±0.06^d^
*Chlorobi*	0^b^	0.02±8.21E−05^a^	0^b^	0^b^
*Chloroflexi*	0^b^	0^b^	0^b^	0.02±7.97E−05^a^
*Cyanobacteria/Chloroplast*	0.07±0.01^b^	0^c^	0^c^	0.49±0.0^a^
*Deinococcus-Thermus*	0^a^	0^a^	0^a^	6.50E−03±4.60E−05^a^
*Firmicutes*	4.42±0.13^c^	8.84±0.18^a^	5.80±0.02^b^	3.40±0.10^d^
*Fusobacteria*	0^b^	0.03±0.01^a^	0^b^	0.02±7.97E−05^a^
*Gemmatimonadetes*	0.04±0.01^b^	1.04±0.01^a^	0.07±0.02^b^	1.22±0.06^a^
*Nitrospira*	0^b^	0.21±0.01^a^	0^b^	0^b^
*Planctomycetes*	0^b^	0^b^	0^b^	6.50E−03±4.60E−05^a^
*Proteobacteria*	92.96±0.17^a^	75.31±0.28^d^	91.45±0.19^b^	82.33±0.22^c^
*Spirochaetes*	0^a^	0^a^	0^a^	6.50E−03±4.60E−05^a^
*Tenericutes*	0^a^	4.10E−03±4.10E−05 ^a^	0^a^	0^a^
TM7	0^b^	0.03±0.01^a^	0^b^	0^b^
unclassified_Bacteria	0.15±0.03^c^	1.90±0.09^a^	0.05±0.01^b^	1.35±0.06^d^

Abbreviations: FI, female-intestine; FR, female-ovary; MI, male-intestine; MR, male-testis.

Abundance with the same letter are not significantly different (P>0.05).

These results suggest that the factor of tissue has more significant influence on the bacterial community structure in *B.minax* than sex.

## Discussion

In the present study we found that the bacterial community of the *B. minax* was very large, with 319–415 OTUs per sample at 97% ID detected. Our results showed that the microbial communities of all samples were dominated by *Proteobacteria*, followed by *Firmicutes* and *Actinobacteria*. Other studies have also found an abundance of *Proteobacteria* in various invertebrates, such as the ground beetles [22], the Lutzomyia Sand Fly [23] and the desert locust, *Schistocerca Gregaria* [14]. On the contrary, bacterial communities associated with certain insect pests, such as termites [24] and bees [25] are more commonly dominated by *Bacteroidetes* and *Firmicutes*. Within *Proteobacteria*, members of the family *Enterobacteriaceae* composed most of the bacterial communities, which is also dominant in the gut of other fruit fly species [5], [16]. The wide distribution of *Enterobacteriaceae* suggested that it has an important function in insects. Although nitrogen is abundant in the atmosphere, paradoxically, it is a limited resource for multicellular organisms [26] and *Enterobacteriaceae* is a member of diazotrophic bacteria, which can help insects fix nitrogen [27], [28]. Furthermore, research has shown that the *Enterobacteriaceae* community within the medfly's gut may have an indirect contribution to host fitness by preventing the establishment or proliferation of pathogenic bacteria [1]. In our study the most prominent species were *Klebsiella*, followed by genus *Citrobacter*. Eyal Ben Ami's study showed that the *K. oxytoca*-enhanced diets significantly shortened the mating latency of the sterile male *Mediterranean* fruit flies [29]. Research from Romero *et al.* revealed that *Citrobacter freundii* stimulated oviposition to the greatest extent and also sustained stable fly development [30]. *Serratia*, another member of *Enterobacteriaceae*, is a pathogen of some other insects [31]. While our data showed that *Serratia* is a common genus in all of our samples. Reports from Fitt [32] and Lloyd [33] have also revealed a similar phenomenon. Some species in this genus can produce chitinase, suggesting their role in insect development [31].

*Firmicutes* was also a major component in both the guts and reproductive organs of the *B. minax*. *Lactococcus* was the most abundant genus found from this phylum which was also found in the Mexican fruit fly [34] and the ant *Solenopsis invicta* [35]. *Actinobacteria* was another major member of the bacterial communities in the reproductive organs in our study. *Actinobacteria* exhibits diverse physiological and metabolic properties, such as the production of extracellular enzymes and the formation of a wide variety of secondary metabolites [36]. The additional species may represent low-abundance taxa present in some or all samples; however, they may become dominant under some special circumstances, as previously reported in coral hosts [37]. Thus, the minor bacteria may act as opportunistic pathogens when their growth and division are out of control.

According to the analysis of the diversity indices, the phylotype composition and the phylogenetic distribution of the pyrosequencing reads, we found that the bacterial communities were significantly different between the gut and the reproductive organs; however, the influence of sex on the composition of the bacterial communities in the fruit fly is minimal. The bacterial communities in the testis and ovary libraries were more diverse than in the gut. This result is in contrast with some previous studies which found that bacteria existed mainly in the midgut and less in the salivary glands and reproductive organs [38], [39]. This variation could be related to the citrus fruit flies' food sources, as the bacterial communities were variable between samples and changed with external factors, such as food sources and surroundings [40]. The *B. minax* larvae only fed on citrus fruits, while the adults fed on honeydew. Thus, these flies had less of a chance of having diverse gut flora. The impact of diet on the composition of the gut microbiota was also described in Colman's report [41].

In summary, our study revealed the composition and diversity of the bacterial communities in the gut and reproductive organs of the citrus fruit fly using 454 - pyrosequencing of 16S rDNA gene amplicons and compared the diversity of the harbored bacterial communities from different sexes and tissues. Among the microbial communities, the most representative species are *Klebsiell*, *Citrobacter, Enterobacter* and *Serratia*. In *B. minax* adults, the reproductive organs have more diverse bacterial communities than the gut. Further studies will be focused on identifying the function of every representative species and establishing whether these species could play important roles in the future as biocontrol agents.

## Materials and Methods

### Ethics Statement

*B. minax* has not been notified under any act or laws and rules of the Government of China for its collection and observation since 2009. No permits were required for collecting the adults since they were obtained from our own land and reared in the laboratory.

### Collection and rearing of insects

The *B. minax* adults were obtained from our experimental field in Zigui country, Hubei province and were reared at 27±1°C and 70–80% relative humidity with a natural photoperiod in the insectary of the Institute of Urban and Horticultural Pests, Huazhong Agricultural University, Wuhan, China. The adults were fed 5% sugar water and sterile water.

### Bactrocera minax dissection

*B. minax* adult samples were surface-sterilized with 75% ethanol for 2–5 min and rinsed three times in sterile water before dissection. The flies were dissected in a plate containing 10 ml sterile phosphate-buffered solution (PBS, pH 7.4) underneath a stereomicroscope. The dissected gut and reproductive organs were transferred separately to a tube containing 1 ml of extract solution (soil fastDNA extraction kit (BioTeke Corporation)) and were homogenized. Each sample comprised three biological replicates and every replicate contained 15 guts or reproductive organs. The homogenate was used for DNA extraction.

### DNA extraction

DNA extraction was performed using the soil fastDNA extraction kit (BioTeke Corporation) following the manufacturer's protocol. Briefly, 5 µl of solution A was added to every tube, and the tubes were vortexed for 1–2 min; then the samples were mixed and incubated in water at 37°C for 10 min. One hundred microliters of solution B was added to very tube, and the tubes were vortexed for 1–2 min; then the samples were mixed and incubated in water at 65°C for 10 min. The samples were spun at 10000 rpm for 10 min, and the supernatants were transferred to new 1.5 ml centrifuge tubes. Then, 1/3 volume of protein precipitating solution was added, and the samples were mixed and placed on ice for 8 min. The samples were then spun at 13000 rpm for 10 min. The supernatant was transferred to a pretreated purification column and spun at a low speed (≤2000 g). The filtrate was collected and a 0.6 volume of isopropanol was added. The samples were mixed and spun at 13000 rpm for 10 min. The supernatant was poured off carefully. The pellet was air-dried for 2 min at room temperature and the DNA sample was resuspended with 30 µl elution buffer.

### 454 pyrosequencing of 16S rDNA gene sequences

We amplified the V3-V4 region of the bacterial 16S rDNA gene to assess the microbial diversity of the flies. The following primers were used for the PCR reaction: 5′-(CCATCTCAT CCCTGCGTGTCTCCGACGACT) XXXXXXXXTACGGRAGGCAGCAG-3′ and reverse 5′- (CCTATCCCCTGTGTGCCTTGGCAGTCTCAG) XXXXXXXX AGGGTATCT- AATCCT-3′. These primers were designed to contain an 8 nt barcode sequence for multiple samples. The adaptor sequences are in parentheses, and Xs represent the barcode sequences. The gene-specific primers 16S343F and 16S798R are underlined. The PCR was performed in triplicate for each sample in a total reaction volume of 20 µl. Each 20 µL reaction mixture contained 0.75 U Takara Ex Taq DNA polymerase, 2 µL 10 X Ex Taq buffer (Mg2+), 1.6 µL dNTP, and 0.4 µL each of the forward and reverse primers, 1 µL of extracted DNA template, 1 µL BSA and up to 20 µL with ddH_2_O. The PCR conditions were the following: initial denaturing at 95°C for 5 min; 30 cycles of 94°C for 30 s, 56°C for 30 s and 72°C for 30 s; and a final extension at 72°C for 10 min. The PCR products were checked with a 2% agarose gel electrophoresis, purified using the AxyPrep DNA gel extraction kit and quantified using the fluorescence quantitative(Qubit 2.0 Fluorometer) with the Qubit dsDNA HS assay kit (Invitrogen, USA). A mixture of the PCR products was prepared by mixing an equal amount of the DNA of the purified 16S amplicons from each sample and then the mixture was cleaned using the Agencourt AMPure XP (Beckman, USA). The mixture was quantified again using the fluorescence quantitative (Qubit 2.0 Fluorometer) with the Qubit dsDNA HS assay kit (Invitrogen, USA) and pyrosequenced on the ROCHE 454 GS FLX platform at Shanghai Hanyubiotech Co., Ltd.

### Data analysis

Raw sequencing reads were quality trimmed according to the flowing requirements: Pyrosequencing reads with ambiguous nucleotides, shorter than 90-nucleotides or without a complete barcode and primer at one end were removed. Qualified reads from each sample were aligned with MUSCLE [42]. A distance matrix was calculated from the alignment with PHYLIP version [43]. This matrix served as an input to DOTUR [44] for clustering the sequences into operational taxonomic units (OTUs) to generate rarefaction curves and to make calculations with the richness and diversity indexes at different dissimilarity levels. The tag pyrosequence reads were classified into different taxonomic groups using RDP classifier [45], [46]. The numbers of representative reads for each OTU determined at 3% dissimilarity at each taxonomic level was counted and the proportions of each group in a sample were calculated. A tree was built using the neighbor-joining method of Clustal_X [47]. The tree was displayed and edited using the Molecular Evolutionary Genetics Analysis (MEGA 5) [48]. A minimum of 75% sequence similarity was applied for assigning the tag sequences into the cluster. The relative abundance and occurrence of tag sequences assigned into clusters were visualized as a heatmap using the JColorGrid [49]. The pyrosequencing data has been submitted to the GenBank database as a file under accession number SRR1531158. The accession numbers of OTU sequences were KJ780722–KJ780731 and KM058914–KM061009.

## Supporting Information

Figure S1 **Dendrogram showing the similarity of bacterial communities from each sample.** The figure was constructed on the basis of tag pyrosequencing data. Abbreviations: FI, female-intestine; FR, female-ovary; MI, male-intestine; MR, male-testis. The numbers 1, 2 and 3 represented the three replicates for each type of sample.
(TIF)

## References

[1] Dillon RJ, Dillon VM (2004) The gut bacteria of insects: nonpathogenic interactions. Annual Review of Entomology 49: 71–92. 10.1146/annurev.ento.49.061802.123416 14651457

[2] Wong CNA, Patrick N, Douglas AE (2011) Low-diversity bacterial community in the gut of the fruitfly *Drosophila melanogaster* . Environmental Microbiology 13(7): 1889–1900. 21631690 10.1111/j.1462-2920.2011.02511.x PMC3495270

[3] Jones RT, Bressan A, Greenwell AM, Fierer N (2011) Bacterial Communities of Two Parthenogenetic Aphid Species Cocolonizing Two Host Plants across the Hawaiian Islands. Applied and environmental microbiology 77(23): 8345–8349. 21965398 10.1128/AEM.05974-11 PMC3233044

[4] Behar A, Yuval B, Jurkevitch E (2008) Gut bacterial communities in the Mediterranean fruit fly (*Ceratitis capitata*) and their impact on host longevity. Journal of Insect Physiology 54(9): 1377–1383. 18706909 10.1016/j.jinsphys.2008.07.011

[5] Jones RT, Sanchez LG, Fierer N (2013) A Cross-Taxon Analysis of Insect-Associated Bacterial Diversity. PLoS ONE 8(4): e61218. 23613815 10.1371/journal.pone.0061218 PMC3628706

[6] Osborne S, Leong Y, O′Neill S, Johnson K (2009) Variation in antiviral protection mediated by different *Wolbachia* strains in *Drosophila simulans* . PLOS Pathogens 5(11): e1000656. 19911047 10.1371/journal.ppat.1000656 PMC2768908

[7] Teixeira L, Ferreira A, Ashburner M (2008) The bacterial symbiont *Wolbachia* induces resistance to RNA viral infections in *Drosophila melanogaster* . Plos Biology 6(12): e2. 10.1371/journal.pbio.1000002 PMC2605931 19222304

[8] Koch H, Schmid HP (2011) Socially transmitted gut microbiota protect bumble bees against an intestinal parasite. Proceedings of the National Academy of Sciences of the United States of America 108: 19288–19292. 22084077 10.1073/pnas.1110474108 PMC3228419

[9] Montllor CB, Maxmen A, Purcell AH (2002) Facultative bacterial endosymbionts benefit pea aphids *Acyrthosiphon pisum* under heat stress. Ecological Entomology 27(2): 189–195.

[10] Santavy DL, Willenz P, Colwell RR (1990) Phenotypic study of bacteria associated with the *Caribbean sclerosponge*, *Ceratoporella nicholsoni* . Applied and Environmental Microbiology 56(6): 1750–1762. 2383012 10.1128/aem.56.6.1750-1762.1990 PMC184505

[11] Pedersen JC, Hendriksen NB (1993) Effect of passage through the intestinal-tract of detritivore earthworms (*Lumbricus spp*.) on the number of selected gram-negative and total bacteria. Biology and Fertility of Soils 16: 227–232.

[12] Eilers H, Pernthaler J, Gloeckner FO, Amann R (2000) Culturability and In Situ Abundance of Pelagic Bacteria from the North Sea. Applied and Environmental Microbiology 66(7): 3044–3051. 10877804 10.1128/aem.66.7.3044-3051.2000 PMC92109

[13] Zhang H, Jackson TA (2008) Autochthonous bacterial flora indicated by PCR-DGGE of 16S rRNA gene fragments from the alimentary tract of *Costelytra zealandica* (*Coleoptera: Scarabaeidae*). Journal of Applied Microbiology 105: 1277–1285. 18713286 10.1111/j.1365-2672.2008.03867.x

[14] Dillon RJ, Webster G, Weightman AJ, Keith CA (2010) Diversity of gut microbiota increases with aging and starvation in the desert locust. Antonie Van Leeuwenhoek 97: 69–77. 19876756 10.1007/s10482-009-9389-5

[15] Ye L, Shao MF, Zhang T, Tong AHY, Lok S (2011) Analysis of the bacterial community in a laboratory-scale nitrification reactor and a wastewater treatment plant by 454-pyrosequencing. Water Research 45: 4390–4398. 21705039 10.1016/j.watres.2011.05.028

[16] Marchini D, Rosetto M, Dallai R, Marri L (2002) Bacteria associated with the oesophageal bulb of the medfly *Ceratitis capitata (Diptera: Tephritidae*). Current Microbiology 44: 120–124. 11815856 10.1007/s00284-001-0061-1

[17] Wang H, Jin L, Zhang H (2011) Comparison of the diversity of the bacterial communities in the intestinal tract of adult *Bactrocera dorsalis* from three different populations. Journal of Applied Microbiology 110(6): 1390–1401. 21395953 10.1111/j.1365-2672.2011.05001.x

[18] Shi ZH, Wang LL, Zhang HY (2012) Low Diversity Bacterial Community and the Trapping Activity of Metabolites from Cultivable Bacteria Species in the Female Reproductive System of the Oriental Fruit Fly, *Bactrocera dorsalis* Hendel (*Diptera: Tephritidae*). International Journal of Molecular Sciences 13: 6266–6278. 22754363 10.3390/ijms13056266 PMC3382823

[19] Zhang HY, Li HY (2012) Photographic guide to key control techniques for citrus disease and insect pests. Beijing: Chinese Agricultural Press.

[20] Lan J, Wan BR, Li KS, Liao GX, Li CR (2009) The attraction effect of fruitfly food lure to Chinese citrus fly. Plant Quarantine 23(2).

[21] Lan J, Zhao Q, Li KS, Li CR (2010) The influences of toxic soils on the emergence of *Bactrocera* (*Tetradacu*) *minax* . Plant Protection 36(1): 159–161.

[22] Jonathan G, Lundgren R, Michael L, Joanne CS (2007) Bacterial Communities within Digestive Tracts of Ground Beetles (*Coleoptera: Carabidae*). Annals of the Entomological Society of America 100(2): 275–282.

[23] Sant'Anna MRV, Darby AC, Brazil RP, Montoya LJ, Dillon VM, et al (2012) Investigation of the Bacterial Communities Associated with Females of Lutzomyia Sand Fly Species from South America. PLoS ONE 7(8): e42531. 22880020 10.1371/journal.pone.0042531 PMC3411800

[24] Xiang H, Xie L, Zhang J, Long YH, Liu N, et al (2012) Intracolonial differences in gut bacterial community between worker and soldier castes of *Coptotermes formosanus* . Insect Science 19: 86–95.

[25] Mohr KI, Tebbe CC (2006) Diversity and phylotype consistency of bacteria in the guts of three bee species (*Apoidea*) at an oilseed rape field. Environmental Microbiology 8(2): 258–272. 16423014 10.1111/j.1462-2920.2005.00893.x

[26] Guerrero DM, Perez F, Conger NG, Solomkin JS, Adams MD, et al (2010) Acinetobacter baumannii-associated skin and soft tissue infections: recognizing a broadening spectrum of disease. Surgical Infection 11(1): 49–57. 10.1089/sur.2009.022 PMC2956563 19788383

[27] Behar A, Yuval B, Jurkevitch E (2005) Enterobacteria-mediated nitrogen fixation in natural populations of the fruit fly *Ceratitis capitata* . Molecular Ecology 14(9): 2637–2643. 16029466 10.1111/j.1365-294X.2005.02615.x

[28] Dixon R, Kahn D (2004) Genetic regulation of biological nitrogen fixation. Nature Reviews Microbiology 2: 621–631. 15263897 10.1038/nrmicro954

[29] Ami EB, Yuval B, Jurkevitch E (2010) Manipulation of the microbiota of mass-reared Mediterranean fruit flies *Ceratitis capitata* (*Diptera: Tephritidae*) improves sterile male sexual performance. International Society for Microbial Ecology 4: 28–37. 10.1038/ismej.2009.82 19617877

[30] Romero A, Broce A, Zurek L (2006) Role of bacteria in the oviposition behaviour and larval development of stable flies. Med Vet Entomol 20: 115–121. 16608496 10.1111/j.1365-2915.2006.00602.x

[31] Grimont F, Grimont PAD (2006) The Genus *Serratia* . Prokaryotes 6: 219–24.

[32] Fitt GP, O′Brien RW (1985) Bacteria associated with four species of *Dacus* (*Diptera: Tephritidae*) and their role in the nutrition of the larvae. Oecologia (Berl) 67: 447–454. 28311582 10.1007/BF00384954

[33] Lloyd AC, Drew RAI, Teakle DS, Hayward AC (1986) Bacteria associated with some *Dacus* species (*Diptera: Tephritidae*) and their host fruit in Queensland. Aust J Biol Sci 39: 361–368.

[34] Kuzina LV, Peloquin JJ, Vacek DC, Miller TA (2001) Isolation and identification of bacteria associated with adult laboratory Mexican fruit flies, *Anastrepha ludens* (*Diptera: Tephritidae*). Current Microbiology 42(4): 290–294. 11178731 10.1007/s002840110219

[35] Ishak HD, Plowes R, Sen R, Kellner K, Meyer E, et al (2011) Bacterial diversity in *Solenopsis invicta* and *Solenopsis geminata* ant colonies characterized by 16S amplicon 454 pyrosequencing. Microbial Ecology 61(4): 821–31. 21243351 10.1007/s00248-010-9793-4

[36] Schrempf H (2001) Recognition and degradation of chitin by *Streptomycetes* . Antonie van Leeuwenhoek 79: 285–289. 11816971 10.1023/a:1012058205158

[37] Venn AA, Loram JE, Trapido RHG, Joyce DA, Douglas AE (2008) Importance of time and place: patterns in abundance of *Symbiodinium* clades A and B in the tropical sea anemone *Condylactis gigantea* . Biological Bulletin 215: 243–252. 19098145 10.2307/25470708

[38] Crotti E, Rizzi A, Chouaia B, Ricci I, Favia G, et al (2010) Acetic acid bacteria, newly emerging symbionts of insects. Applied and Environmental Microbiology 76(21): 6963–6970. 20851977 10.1128/AEM.01336-10 PMC2976266

[39] Rani A, Sharma A, Rajagopal R, Adak T, Bhatnagar RK (2009) Bacterial diversity analysis of larvae and adult midgut microflora using culture-dependent and culture-independent methods in lab-reared and field-collected *Anopheles stephensi*-an Asian malarial vector. BMC Microbiology 9: 96. 19450290 10.1186/1471-2180-9-96 PMC2698833

[40] Michael BY, Jurkevitch E, Yuval B (2008) Effect of bacteria on nutritional status and reproductive success of the Mediterranean fruit fly *Ceratitis capitata* . Physiological Entomology 33(2): 145–154.

[41] Colman DR, Toolson EC, Takacs VCD (2012) Do diet and taxonomy influence insect gut bacterial communities? Molecular Ecology 21: 5124–5137. 22978555 10.1111/j.1365-294X.2012.05752.x

[42] Edgar RC (2004) MUSCLE: multiple sequence alignment with high accuracy and high throughput. Nucleic Acids Research 32(5): 1792–1797. 15034147 10.1093/nar/gkh340 PMC390337

[43] Felsenstein J (2005) PHYLIP (Phylogeny Inference Package) version 3.6. Department of Genome Sciences, University of Washington, Seattle Available: http://evolution.genetics.washington.edu/phylip.html. Accessed 2014 Aug 25..

[44] Schloss PD, Handelsman J (2005) Introducing DOTUR, a computer program for defining operational taxonomic units and estimating species richness. Applied and Environmental Microbiology 71: 1501–1506. 15746353 10.1128/AEM.71.3.1501-1506.2005 PMC1065144

[45] Cole JR, Wang Q, Cardenas E, Fish J, Chai B, et al (2008) The Ribosomal Database Project: improved alignments and new tools for rRNA analysis. Nucleic Acids Research 37: 141–145. 10.1093/nar/gkn879 PMC2686447 19004872

[46] Hua WY (2010) The method study of the application of 454 pyrosequencing on microbial community analysis. Shanghai Jiao Tong University Available: http://epub.cnki.net/kns/brief/default_result.aspx. Accessed 2010 Mar 1..

[47] Thompson JD, Gibson TJ, Plewniak F, Jeanmougin F, Higgins DG (1997) The CLUSTAL_X windows interface: Flexible strategies for multiple sequence alignment aided by quality analysis tools. Nucleic Acids Research 25: 4876–4882. 9396791 10.1093/nar/25.24.4876 PMC147148

[48] Tamura K, Peterson D, Peterson N, Stecher G, Nei M, et al (2011) MEGA5: molecular evolutionary genetics analysis using maximum likelihood, evolutionary distance, and maximum parsimony methods. Mol. Biol. Evol 28(10): 2731–2739. 21546353 10.1093/molbev/msr121 PMC3203626

[49] Joachimiak MP, Weisman JL, May BCh (2006) JColorGrid: software for the visualization of biological measurements. BMC Bioinformatics 7: 225–229. 16640789 10.1186/1471-2105-7-225 PMC1479842

