# Supplementary figures and images for: Bacterial Communities in the Gut and Reproductive Organs of Bactrocera minax (Diptera: Tephritidae) Based on 454 Pyrosequencing
Source: PLoS One. 2014 Sep 12;9(9):e106988. doi: 10.1371/journal.pone.0106988 (PMC4162550; doi:10.1371/journal.pone.0106988)

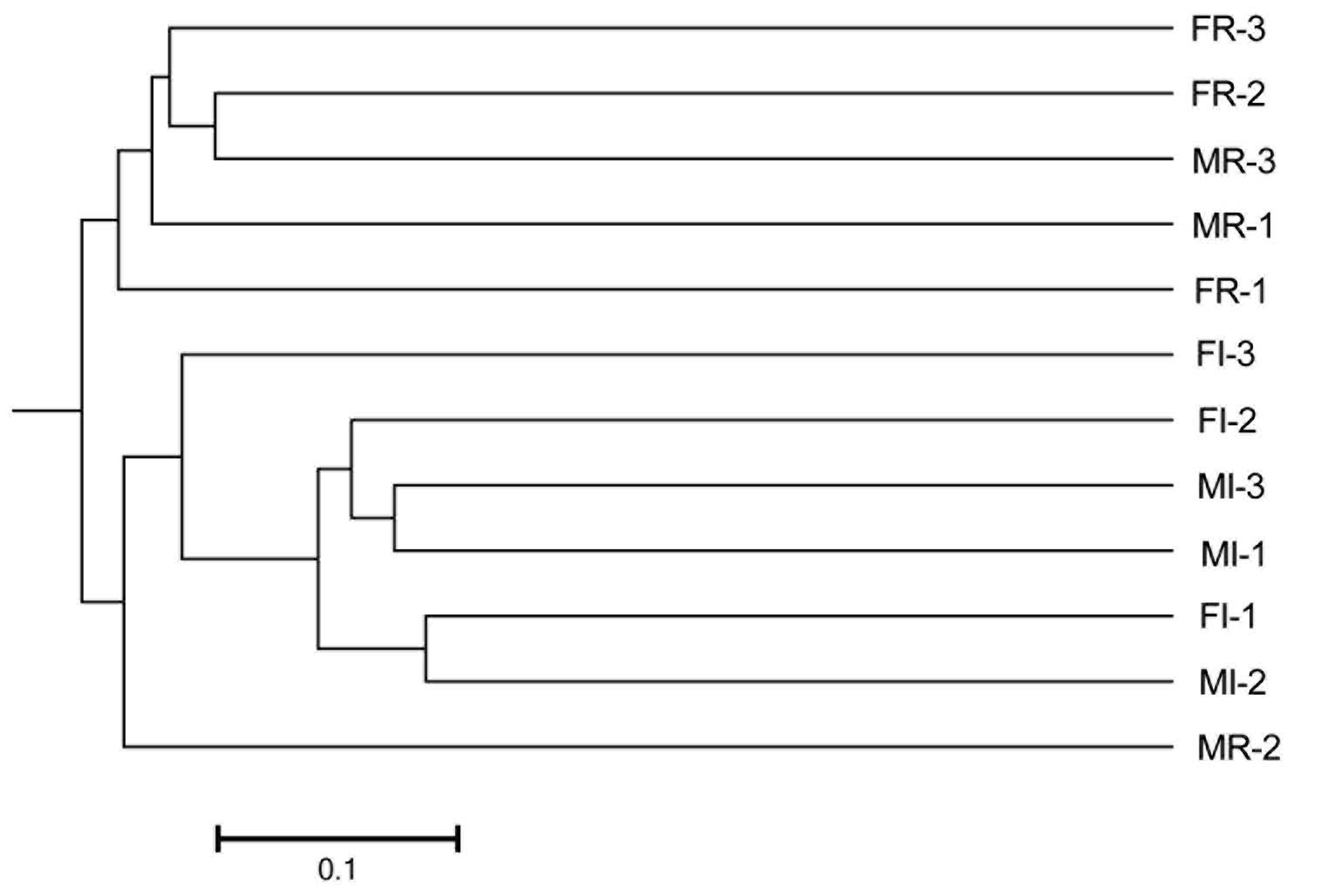

Supplement: Figure S1 — Dendrogram showing the similarity of bacterial communities from each sample. The figure was constructed on the basis of tag pyrosequencing data. Abbreviations: FI, female-intestine; FR, female-ovary; MI, male-intestine; MR, male-testis. The numbers 1, 2 and 3 represented the three replicates for each type of sample. (TIF) [file pone.0106988.s001.tif]
